# A deletion containing a CTCF-element in intron 8 of the *Bbs7* gene is partially responsible for juvenile obesity in the Berlin Fat Mouse

**DOI:** 10.1007/s00335-021-09938-5

**Published:** 2021-12-15

**Authors:** Florian Krause, Kourosh Mohebian, Manuel Delpero, Deike Hesse, Ralf Kühn, Danny Arends, Gudrun A. Brockmann

**Affiliations:** 1 Albrecht Daniel Thaer-Institute for Agricultural and Horticultural Sciences, Humboldt-Universität zu Berlin, Unter den Linden 6, 10099 Berlin, Germany; 2 Max-Delbrück-Center for Molecular Medicine in the Helmholtz Association, Robert-Rössle-Str. 10, 13125 Berlin, Germany

## Abstract

The Berlin Fat Mouse Inbred (BFMI) line is a model for juvenile obesity. Previous studies on crosses between BFMI and C57Bl/6N (B6N) have identified a recessive defect causing juvenile obesity on chromosome 3 (*jObes1*). *Bbs7* was identified as the most likely candidate gene for the observed effect. Comparative sequence analysis showed a 1578 bp deletion in intron 8 of *Bbs7* in BFMI mice. A CTCF-element is located inside this deletion. To investigate the functional effect of this deletion, it was introduced into B6N mice using CRISPR/Cas9. Two mice containing the target deletion were obtained (B6N *Bbs*7^emI8∆1^ and *Bbs7*^emI8∆2^) and were subsequently mated to BFMI and B6N to generate two families suitable for complementation. Inherited alleles were determined and body composition was measured by quantitative magnetic resonance. Evidence for a partial complementation (13.1–15.1%) of the *jObes1* allele by the CRISPR/Cas9 modified B6N *Bbs*7^emI8∆1^ and *Bbs7*^emI8∆2^ alleles was found. Mice carrying the complementation alleles had a 23–27% higher fat-to-lean ratio compared to animals which have a B6N allele (*P*_(Bbs7emI8∆1)_ = 4.25 × 10^–7^; *P*_(Bbs7emI8∆2)_ = 3.17 × 10^–5^). Consistent with previous findings, the recessive effect of the BFMI allele was also seen for the B6N *Bbs*7^emI8∆1^ and *Bbs7*^emI8∆2^ alleles. However, the effect size of the B6N *Bbs7*^*emI8*∆1^ and *Bbs7*^*emI8*∆2^ alleles was smaller than the BFMI allele, and thus showed only a partial complementation. Findings suggest additional variants near *Bbs7* in addition to or interacting with the deletion in intron 8.

**Supplementary Information:**

The online version contains supplementary material available at 10.1007/s00335-021-09938-5.

## Introduction

The Berlin Fat Mouse Inbred line (BFMI) is a model for juvenile obesity. It was selected for high body fat content under standard diet (Wagener et al. 2006). BFMI mice have several features of the metabolic syndrome such as impaired lipid metabolism and reduced insulin sensitivity (Meyer et al. 2009; Hantschel et al. 2011; Heise et al. 2016). The BFMI mice develop obesity between 3 and 8 weeks (Wagener et al. 2006). Already under a standard breeding diet, BFMI males and females at 10 weeks have on average 8.4 times more body fat mass than C57BL/6NCrl (B6N) reference mice (Wagener et al. 2006). A region on chromosome 3 (*jObes1* locus) was identified that explains 40% of the variation of fat mass in an F2 population from a cross between the obese BFMI and the lean B6N mouse line (Neuschl et al. 2010). Further fine mapping in an advanced intercross population generated from the original mapping population reduced the confidence interval to ~ 370 kb. Furthermore, complementation tests with different knockout mice in the target region revealed the Bardet Biedl syndrome 7 (*Bbs7*) gene as the most likely candidate for the juvenile obesity phenotype in BFMI mice (Arends et al. 2016).

Sequence comparisons between the BFMI and B6N mouse lines in the *Bbs7* gene region showed a deletion of 1578 bp in intron 8 of the *Bbs7* gene (3:36,599,424–36,602,828) in BFMI mice (available from NCBI Sequence Read Archive (SRA) PRJNA717237). This deletion also leads to the loss of a binding site for the CCCTC-binding factor (CTCF). This CTCF-element in intron 8 was initially found by mapping DNase1 hypersensitive sites and by CTCF ChIP-Seq experiments in embryonic stem cells (Stadler et al. 2011). Such a CTCF-element may act as an insulator that is able to block the activity of enhancers through modification of the 3D DNA structure or act as a promoter activator or repressor (Phillips and Corces 2009). The binding of a CTCF-insulator binding protein was verified via the ChIP-Seq method (Chen et al 2008). ChIP-Seq can unveil a high range of binding sites for effectors such as CTCF-element, as it can recognize binding sites which exist at repetitive elements (Chen et al. 2008). Repetitive elements also occur around the CTCF-element in the deleted *Bbs7* intron region. Re-mapping of the experimental data of Chen et al. in embryonic stem cells to the current genome of the mouse showed that CTCF binds indeed in intron 8 to the region of the deletion in the BFMI line (Ferrai et al. 2017).

In this study, the targeted generation of the BFMI deletion in intron 8 of *Bbs7* using CRISPR/Cas9 (Ran et al. 2013) combined with complementation tests were used to identify the genetic effect of the deleted intron 8 region of *Bbs7* on the development of obesity.

## Materials and methods

### Mouse populations

The Berlin Fat Mouse Inbred line 860-12 (BFMI) was used as our model for juvenile obesity. The line C57BL/6NCrl (B6N, Charles River Laboratories, Sulzfeld, Germany) served as lean control. These two lines have been used before for mapping the juvenile obesity QTL on mouse chromosome 3 (Neuschl et al. 2010; Arends et al. 2016).

### CRISPR/Cas9

Whole genome sequencing showed a deletion of 1578 bp (3:36,599,424–36,602,828) in intron 8 of *Bbs7* in BFMI mice. This deletion was generated on a B6N background using CRISPR/Cas9 nuclease and a pair of single (sg) guide-RNAs in single-cell mouse embryos as described in detail in (Wefers et al. 2017) and (Brandl et al. 2015). Consistent with the CRISPR/Cas9 endonuclease effect differences with respect to the strand breakpoints resulted in deletions of different lengths.

Briefly, Cas9 mRNA was prepared in a single step by in vitro transcription from plasmid pCAG-Cas9-162A linearized with AsiSI, AscI, and XbaI, using the mMessage mMachine T7 Ultra kit (Life Technologies, Ambion, AM1345, Life Technologies, Carlsbad, USA) (omitting the polyadenylation step). Cas9-162A mRNA was isolated using the Oligotex mRNA mini kit (cat No. 70022, Qiagen, Hilden, Germany). To produce the templates for sgRNA in vitro transcription, sgBbs7 were amplified by PCR from guide RNA containing plasmids. As target sites, we selected two 20 nucleotide sequences located upstream of the deleted DNA segment (sgBbs7-A1: CCACTGTATAGGGCGACCAC, sgBbs7-A2: AGGAGTGCTTGCCACTGTAT) and two sequences located downstream (sgBbs7-B1: CTACATAAAGAAGAGAGGTT, sgBbs7-B2: TAGTCTGTAAATCTCTTCAG).

1 µg template DNA was used for in vitro transcription using the Megashortscript kit (Ambion, cat No. AM1354) followed by the MEGAclear kit (Ambion, cat No. AM1908) for RNA purification. RNAs and targeting vectors were diluted in microinjection buffer (10 mM Tris, 0.1 mM EDTA, pH 7.2) to the indicated working concentrations, filtrated through a centrifugal filter (Ultrafree, PFTE, Millipore, cat. no. UFC30LG25) and stored in single-use aliquots at − 80 °C. For microinjections, zygotes were obtained by mating of C57BL/6 N males with super-ovulated C57BL/6 N females (Charles River, Sulzbach, Germany). Injected zygotes were transferred into pseudo-pregnant NMRI female mice to obtain live pups.

**Fig. 1:**
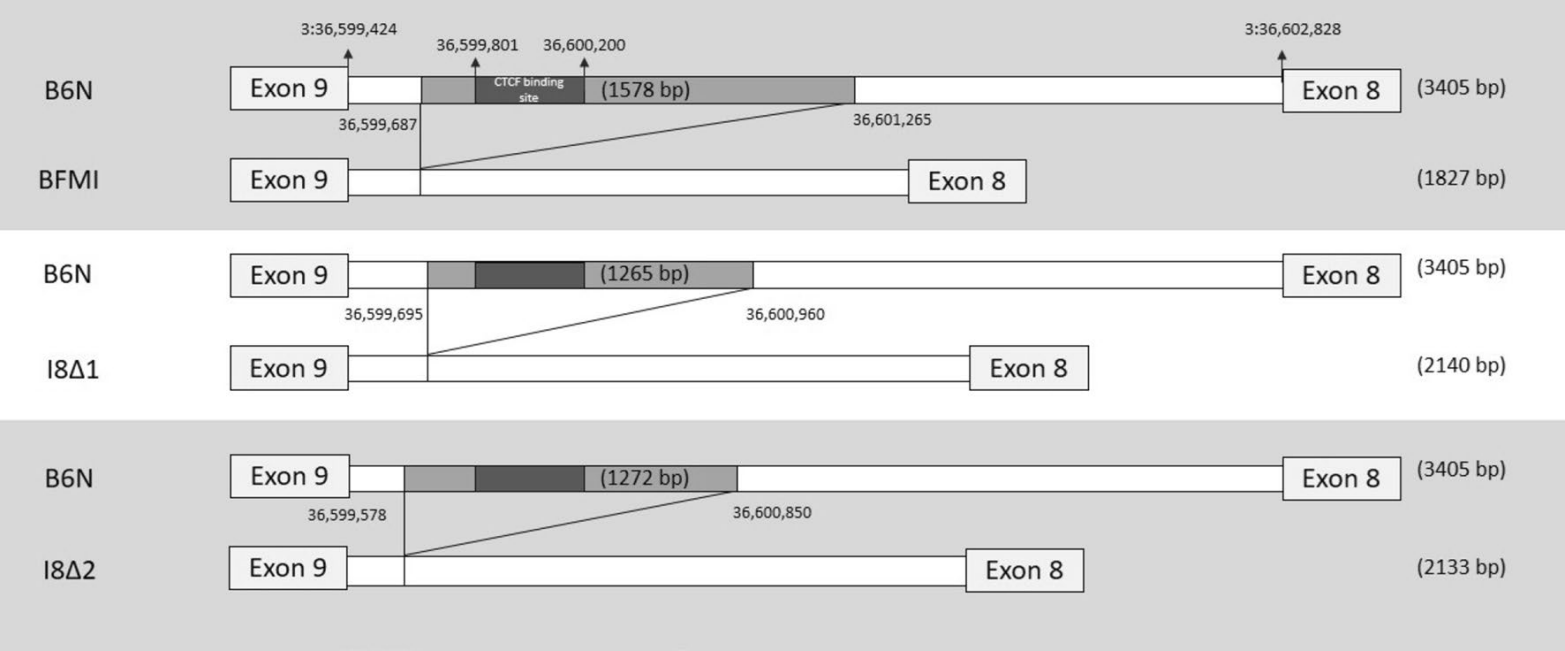
Deletions in the intron 8 of *Bbs7*. Comparison of B6N (reference) to the naturally occurring deletion in the BFMI (top), and the two artificial CRISPR/Cas9 introduced deletions I8∆1 and I8∆2. Please, note that the *Bbs7* gene is on the negative strand, as such Exon 9 is in front of Exon 8

Sanger sequencing was performed to identify the exact DNA-sequence breakpoints (Fig. 1). All positions mentioned in this paper are based on GRCm38.p6 (Ensembl release 102).

The modified mouse lines were named C57BL/6N-*Bbs7*^emI8∆*^, where emI8∆* refers to an endonuclease-mediated deletion of a specific part of intron 8. Two male mice with partial deletions in intron 8 of the *Bbs7* gene, comparable to the BFMI allele, were generated. These mice were used as founders for two separate and independent pedigrees. In each pedigree C57BL/6N-*Bbs7*^emI8∆*^ founder mice were initially crossed with BFMI and B6N mice. Subsequent generations were produced by mating of siblings. We named the families with the obtained deletions I8∆1 (995 bp, 3:36,599,695–36,600,690) and I8∆2 (1,272 bp; 3:36,599,578–36,600,850). Both C57BL/6N-*Bbs7*^emI8∆*^ mice and BFMI (1,578 bp; 36,599,687–36,601,265) share the deletion in intron 8 between 36,599,695 and 36.600,850 (Fig. 1). Two independent families were used since complementation in one of the two might allow to fine map which part of the deletion might be causal. All deletions caused the loss of a CTCF-binding site located at 3:36,599,801–36,600,200. Both male and female animals were included in the analysis.

### Husbandry conditions

All mice were maintained under conventional conditions and a 12:12 h light:dark cycle (lights on from 6 a.m. to 6 p.m.) at a temperature of 22 ± 2 °C. Animals had ad libitum access to food and water. The mice were fed with a rodent standard diet (V1534-000 ssniff R/M-H; Ssniff Spezialdiäten GmbH, Soest, Germany).

### Phenotyping

Total fat mass and total lean mass were determined in non-anesthetized male and female mice at 10 weeks by quantitative magnetic resonance (QMR) using the EchoMRI whole body magnetic resonance analyzer (Echo Medical Systems, Houston, TX, USA) (Wagener et al. 2006). QMR measurements were performed in duplicates and the mean was used for further analyses. The ratio of total fat to total lean mass at 10 weeks (fat-to-lean ratio) was used to characterize the obese phenotype. Generations 7 to 14 of both independent families (I8∆1 and I8∆2) were analyzed in this paper for complementation, see supplemental Table 1 for an overview of numbers of animals (males/females) in each generation.

### Genotyping

DNA was extracted from ear punches. Since three alleles occurred in our pedigrees, we generated a multiplex PCR system that allowed us to simultaneously genotype the BFMI and B6N alleles and the deletion alleles I8∆1 or I8∆2 (Supplementary Table 2). Using two forward and one reverse primer, we could distinguish all three alleles within each pedigree (BFMI, B6N, pedigree-specific CRISPR/Cas9 deleted alleles I8∆1 or I8∆2).

### Data analysis and complementation tests

Phenotype and genotype data were analyzed using the R Project for Statistical Computing v4.0.2 (The R Development Core Team 2005). Phenotype measurements were adjusted for generation and sex since these external factors showed a significant effect on the phenotype. We performed an ANOVA test with the general linear model (corrected for sex and generation) to identify significant differences of the fat-to-lean ratio between genotype classes. Subsequently, a Welch’s *t*-test was used for pairwise group comparison. Differences between genotype classes were considered statistically significant at *P* < 0.05.

## Results

### Covariates (sex, generation, litter size)

A linear model was used to investigate the influence of sex, generation, and litter size on the phenotype. The most significant effect was sex (*P*_(I8∆1)_ = 2.20 × 10^–16^; *P*_(I8∆2)_ = 5.95 × 10^–14^) followed by the generation effect (*P*_(I8∆1)_ = 0.066; *P*_(I8∆2)_ = 0.002). Litter size showed no significant effect on the phenotype in both families (*P*_(I8∆1)_ = 0.158; *P*_(I8∆2)_ = 0.453). As such, we corrected the fat-to-lean ratio for sex and generation. The adjusted fat-to-lean ratio was used in further complementation testing.

### Family 1 (I8∆1)

The I8∆1 allele was tested for complementation with the BFMI allele. B6N allele carriers were used as lean reference. Homozygous B6N carriers were the smallest genotype group (8 animals; mean: 0.112 ± 0.048). However, there was no difference to the other two genotypes carrying one B6N allele (*P*_(B6N/B6N vs. BFMI/B6N)_ = 0.879; *P*_(B6N/B6N vs. I8∆1/B6N)_ = 0.404). By taking this into account, combined with the knowledge that the effect is a recessive effect, these genotypes were merged (x/B6N) to create a larger reference panel consisting of 70 animals (mean: 0.096 ± 0.035).

The two complementing genotypes BFMI/I8∆1 and I8∆1/I8∆1 also showed no significant difference (*P*_(I8∆1/I8∆1 vs. BFMI/I8∆1)_ = 0.971) in fat-to-lean ratio and were also merged into a single complementation group (y/I8∆1) consisting of 440 animals (mean: 0.122 ± 0.047). The positive control group (BFMI/BFMI) of family 1 (I8∆1) contains 31 animals.

**Fig. 2:**
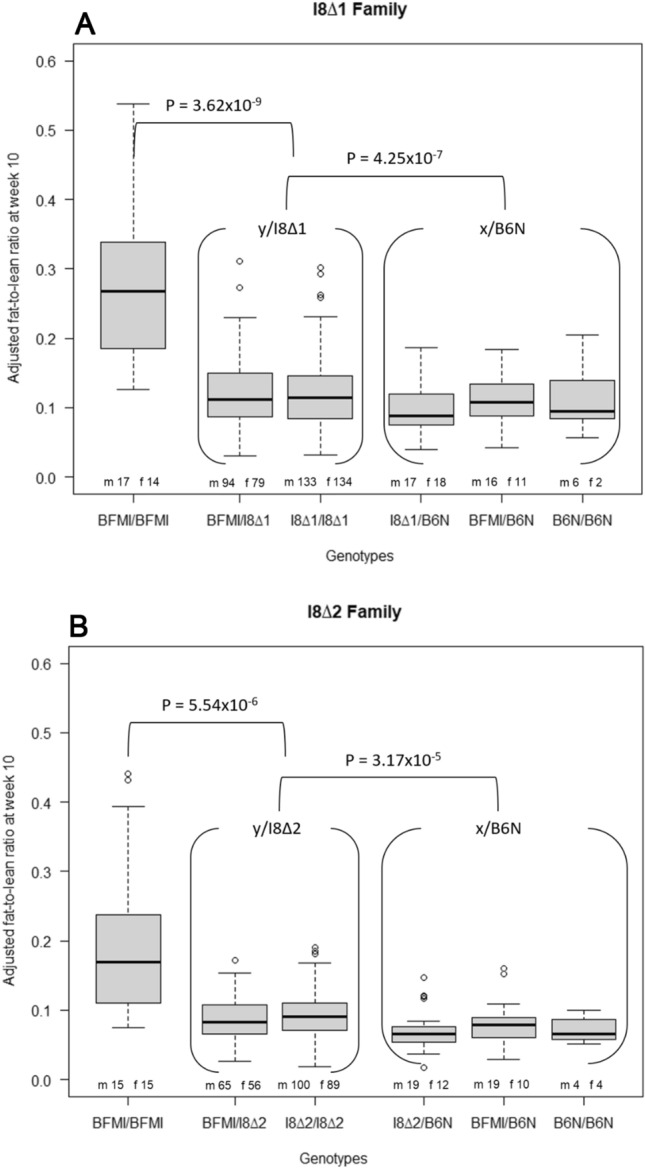
**a** 541 animals of generations 7–14 were analyzed in pedigree I8∆1. The gender breakdown is given on top of the *x*-axis for every genotype (*m* males; *f* females). The phenotype on the *y*-axis was corrected for sex and generation. The homozygous BFMI genotype showed the highest fat-to-lean ratio of 0.268 ± 0.102. The y/I8∆1 (grouping of I8∆1/I8∆1 and BFMI/I8∆2) showed a fat-to-lean ratio of 0.122 ± 0.047, which was significantly lower (*P*_(BFMI/BFMI vs. y/I8∆1)_ = 3.62 × 10^–9^) than in BFMI homozygous mice, but also significantly higher significant (*P*_(y/I8∆1 vs. x/B6N)_ = 4.25 × 10^–7^) than genotypes of the reference group x/B6N (0.096 ± 0.035) (x/B6N is the grouping of B6N/B6N, BFMI/B6N, and I8∆1/B6N). The effect of the I8∆1 allele is 15.1% of the BFMI allele. **b** 408 mice of generation 7–14 were analyzed in pedigree I8∆2. The phenotype on the *y*-axis was corrected for sex and generation. The homozygous BFMI genotype showed the highest fat-to-lean ratio of 0.204 ± 0.112. The y/I8∆2 complementation group showed a fat-to-lean ratio of 0.091 ± 0.032, which was significantly lower (*P*_(BFMI/BFMI vs. y/I8∆2)_ = 5.54 × 10^–6^) than in the BFMI homozygous mice, but significantly higher (*P*_(y/I8∆2 vs. x/B6N)_ = 3.17 × 10^–5^) than genotypes containing the B6N allele (x/B6N) with a fat-to-lean ratio of 0.074 ± 0.028. The effect of the I8∆2 allele is 13.1% of the BFMI allele

Complementation was investigated by comparing the y/I8∆1 group to the positive control (BFMI/BFMI) and the negative control (x/B6N). This comparison showed a significant (*P*_(y/I8∆1 vs. x/B6N)_ = 4.25 × 10^–7^) increase in fat-to-lean ratio from 0.096 for x/B6N animals to 0.122 for y/I8∆1 animals. However, homozygous BFMI (mean: 0.268 ± 0.102) animals showed a significantly (*P*_(BFMI/BFMI vs. y/I8∆1)_ = 3.62 × 10^–9^) higher fat-to-lean ratio compared to the y/I8∆1 group, pointing to a partial complementation (Fig. 2a, Supplementary Table 3).

The effects size, relative to the x/B6N group, showed that the I8∆1 allele increases the fat-to-lean ratio by 0.026, while the homozygous BFMI animals showed an increase of 0.172. This indicates that the I8∆1 allele explains around 15.1% of the BFMI effect.

### Family 2 (I8∆2)

Results from family 2 verify the results obtained in family 1. However, it should be noted that the overall fat-to-lean ratio in family 2 is significantly lower than in family 1 (fat-to-lean_(family2 vs. family1)_ =  − 23.6%, *P*_(family2 vs. family 1)_ = 5.68 × 10^–13^).

Similar to family 1, we also observed no difference between animals carrying one or more B6N alleles (*P*_(B6N/B6N vs. BFMI/B6N)_ = 0.428; *P*_(B6N/B6N vs. I8∆2/B6N)_ = 0.953). Also, for the complementation group no difference was observed (*P*_(I8∆2/I8∆2 vs. BFMI/I8∆2)_ = 0.146). Merging of groups led to a sample size of 68 animals in the x/B6N group and 310 animals in the y/I8∆2 group. Furthermore, the same pattern as in family 1 was seen: x/B6N was the group with the lowest fat-to-lean ratio (0.074 ± 0.028), BFMI/BFMI showed the highest fat-to-lean ratio (0.204 ± 0.112), and a significant increase of the y/I8∆2 group (0.091 ± 0.032) compared to the x/B6N group (*P*_(y/I8∆2 vs. x/B6N)_ = 3.17 × 10^–5^) was observed (Supplementary Table 4). The I8∆2 allele showed a 13.1% partial complementation of the BFMI allele, an effect very similar to the I8∆1 allele.

The uncorrected data are also shown in the supplemental files (Supplemental Figs. 1 and 2).

## Discussion

Both of the CRISPR/Cas9 modified mice and the BFMI mouse share the loss of a region in intron 8 of *Bbs7* between 36,599,695 and 3,600,960 base pairs on chromosome 3 containing a CTCF-element. The deletion of a CTCF-element is known to affect the 3D chromosomal structure (Phillips and Corces 2009) and as a result changes the accessibility of transcription factor binding sites (Ohlsson et al. 2001). Previously, it was observed that the *Bbs7* expression in BFMI mice is significantly reduced compared to the expression of *Bbs7* in B6N mice (Arends et al. 2016). Previous experiments on *Bbs7* knockout mice showed obesity as one of the phenotypes observed in these mice (Zhang et al. 2013). The deleted CTCF-element in intron 8 in BFMI, I8∆1, and I8∆2 may be responsible for reducing *Bbs7* transcription levels, which might cause obesity by influencing fat deposition.

Since we observed only a partial complementation in the I8∆1 and I8∆2 families, one (or more) other variants need to be involved in causing the juvenile obesity phenotype of BFMI mice. Sequence data provided evidence for additional sequence variation between BFMI and B6N inside the *jObes1* locus. Eventually, one of the other sequence variants in BFMI in addition to the deletion in intron 8 is required for full complementation of the BFMI allele effect. These additional sequence variants did not occur in the CRISPR/Cas9 modified B6N mice. The additional sequence variants in BFMI are likely in close proximity to *Bbs7*, since otherwise recombinations would have caused BFMI variants in the background of the modified B6N mice in some animals of the I8∆1, and I8∆2 families. This would have led to a full complementation for some of the mice, which was not observed in our study. The additional sequence variants were not tested in this study. Therefore, we cannot exclude variants outside of the *jObes1* locus that could affect or modulate the juvenile obesity phenotype in BFMI mice directly or in interaction with the deleted region in intron 8 of *Bbs7*.

Several other mouse lines harbor a similar deletion found in BFMI mice. Some of these were previously investigated for complementation (NZO and AKR/J) (Arends et al. 2016). No complementation with NZO and AKR/J was detected in the 2016 study, and there it was concluded that the intron 8 deletion was not causal for the juvenile obesity. With the current finding of a partial complementation by I8∆1 and I8∆2, we revisit the intron 8 effect, and now show that part of the juvenile obesity seen in BFMI mice can be explained by the deletion in intron 8. The previous study missed this partial complementation effect since the sample sizes used were only suitable for investigating full complementation.

## Conclusion

We show that various partial deletions (I8∆1 and I8∆2) in intron 8 of *Bbs7* are able to partially complement the BFMI allele at the *jObes1* locus, seen by an increase in the fat-to-lean ratio. Both I8∆1 and I8∆2 alleles show a recessive effect on the fat-to-lean ratio similar to the BFMI allele. The strengths of complementation of the I8∆1 and I8∆2 alleles are similar. By aligning the three deletions (I8∆1, I8∆2, and BFMI) we found the only common feature shared between the three deletions was a CTCF-element. Additionally, we conclude that another sequence variant at the *jObes1* locus in BFMI is essential to show the full effect on juvenile obesity as seen in BFMI mice. This is because (A) we see only partial complementation meaning another variant should be present, and (B) we know this variant needs to be close to the deletion otherwise recombinations in the two pedigrees would have caused around 50% of offspring with the complementation genotypes to show full complementation due to inheritance of the second factor in the genetic background.

## Supplementary Information

Below is the link to the electronic supplementary material.

Supplementary file1 (DOCX 156 kb)

## Data Availability

Scripts and raw data are available at https://github.com/FlorianKrause/Bbs7-complementation.
