# Supplementary material for: A deletion containing a CTCF-element in intron 8 of the Bbs7 gene is partially responsible for juvenile obesity in the Berlin Fat Mouse
Source: Mamm Genome. 2021 Dec 15;33(3):465–70. doi: 10.1007/s00335-021-09938-5 (PMC9360062; doi:10.1007/s00335-021-09938-5)

Supplementary Information

A deletion containing a CTCF-element in intron 8 of the *Bbs7* gene is partially responsible for juvenile obesity in the Berlin Fat Mouse

Mammalian Genome

*Florian Krause ^1,*^, Kourosh Mohebian ^1,*^, Manuel Delpero ^1^, Deike Hesse ^1^, Ralf Kühn ^2^, Danny Arends ^1^, Gudrun A. Brockmann ^1,^*^†^

^1^ Albrecht Daniel Thaer-Institut für Agrar- und Gartenbauwissenschaften, Humboldt-Universität zu Berlin, Invalidenstraße 42, D-10115 Berlin, Germany

^2^ Max-Delbrück-Center for Molecular Medicine in the Helmholtz Association, Robert-Rössle-Str. 10, D-13125 Berlin, Germany

* First two authors contributed equally

† Corresponding author

**Address for correspondence:**

Gudrun A. Brockmann

Animal Breeding Biology and Molecular Genetics

Humboldt-Universität zu Berlin,

Invalidenstraße 42,

D-10115 Berlin, Germany

Phone: 0049 30 2093 49872
Fax: 0049 30 2093 6397
E-mail: [gudrun.brockmann@agrar.hu-berlin.de](mailto:gudrun.brockmann@agrar.hu-berlin.de)

Table 1: Animals (males/females) in each generation

| Generation | Family 1 (I8Δ1) | | Family 2 (I8Δ2) | |
| --- | --- | --- | --- | --- |
|  | males | females | males | females |
| 7 | 44 | 44 | 20 | 23 |
| 8 | 40 | 42 | 9 | 9 |
| 9 | 41 | 38 | 26 | 30 |
| 10 | 41 | 26 | 27 | 21 |
| 11 | 27 | 32 | 36 | 30 |
| 12 | 29 | 29 | 37 | 25 |
| 13 | 38 | 28 | 25 | 20 |
| 14 | 23 | 19 | 42 | 28 |

Table 2: Designed primers for genotyping

| Deletion | Primer | Primer sequence |
| --- | --- | --- |
| I8Δ1 |  |  |
|  | 1^st^ Fwd | GACACTGAGGAAAACCTGAATGA |
|  | 2^nd^ Fwd | AGCCAGACAAAGTACACATGA |
|  | rev | GCCCTTTGAAACCTGTGCTA |
| I8Δ2 |  |  |
|  | 1^st^ Fwd | GACACTGAGGAAAACCTGAATGA |
|  | 2^nd^ Fwd | CCAAAGCCACACTGACTGTC |
|  | rev | GCCCTTTGAAACCT TGCTA |

Table 3: Means, SDs, and P-values of Family 1 (I8Δ1) for the adjusted fat-to-lean ratio at week 10 of age

|  | Mean (SD) | I8∆1/BFMI | I8∆1/I8∆1 | I8∆1/B6N | BFMI/B6N | B6N/B6N |
| --- | --- | --- | --- | --- | --- | --- |
| BFMI/BFMI | 0.268 (0.102) | 4.51x10^-9^ | 4.85x10^-9^ | 1.11x10^-10^ | 3.81x10^-10^ | 7.37x10^-7^ |
| I8∆1/BFMI | 0.121 (0.047) |  | 0.971 | 0.001 | 0.142 | 0.646 |
| I8∆1/I8∆1 | 0.121 (0.047) |  |  | 0.121 | 0.121 | 0.637 |
| I8∆1/B6N | 0.097 (0.035) |  |  |  | 0.148 | 0.404 |
| BFMI/B6N | 0.110 (0.034) |  |  |  |  | 0.879 |
| B6N/B6N | 0.112 (0.048) |  |  |  |  |  |
| y/I8∆1 | 0.122 (0.047) |  |  |  |  |  |
| x/B6N | 0.096 (0.035) |  |  |  |  |  |
|  |  |  |  |  |  |  |
|  | y/I8∆1 | x/B6N |  |  |  |  |
| BFMI/BFMI | 3.62x10^-9^ | 4.45x10^-11^ |  |  |  |  |
| y/I8∆1 |  | 4.25x10^-7^ |  |  |  |  |

Table 4: Means, SDs, and P-values of Family 2 (I8Δ2) for the adjusted fat-to-lean ratio at week 10 of age

|  | Mean (SD) | I8∆2/BFMI | I8∆2/I8∆2 | I8∆2/B6N | BFMI/B6N | B6N/B6N |
| --- | --- | --- | --- | --- | --- | --- |
| BFMI/BFMI | 0.204 (0.112) | 3.60x10^-6^ | 7.42x10^-6^ | 3.97x10^-7^ | 5.53x10^-7^ | 2.48x10^-7^ |
| I8∆2/BFMI | 0.088 (0.031) |  | 0.146 | 0.006 | 0.148 | 0.043 |
| I8∆2/I8∆2 | 0.093 (0.032) |  |  | 0.020 | 0.020 | 0.011 |
| I8∆2/B6N | 0.071 (0.028) |  |  |  | 0.336 | 0.953 |
| BFMI/B6N | 0.079 (0.030) |  |  |  |  | 0.428 |
| B6N/B6N | 0.072 (0.018) |  |  |  |  |  |
| y/I8∆2 | 0.091 (0.032) |  |  |  |  |  |
| x/B6N | 0.074 (0.028) |  |  |  |  |  |
|  |  |  |  |  |  |  |
|  | y/I8∆2 | x/B6N |  |  |  |  |
| BFMI/BFMI | 5.54x10^-6^ | 2.98x10^-7^ |  |  |  |  |
| y/I8∆2 |  | 3.17x10^-5^ |  |  |  |  |

**Supplementary Figure 1: Uncorrected fat-to-lean ratios of Family 1 (I8Δ1) and Family 2 (I8Δ2) separated into males and females for 10-week-old mice.** No correction was done for generation and litter size, already in the raw data the complementation group (BFMI/I8Δ*) is showing a higher fat-to-lean ratio compared to the negative control (I8Δ*/B6N).


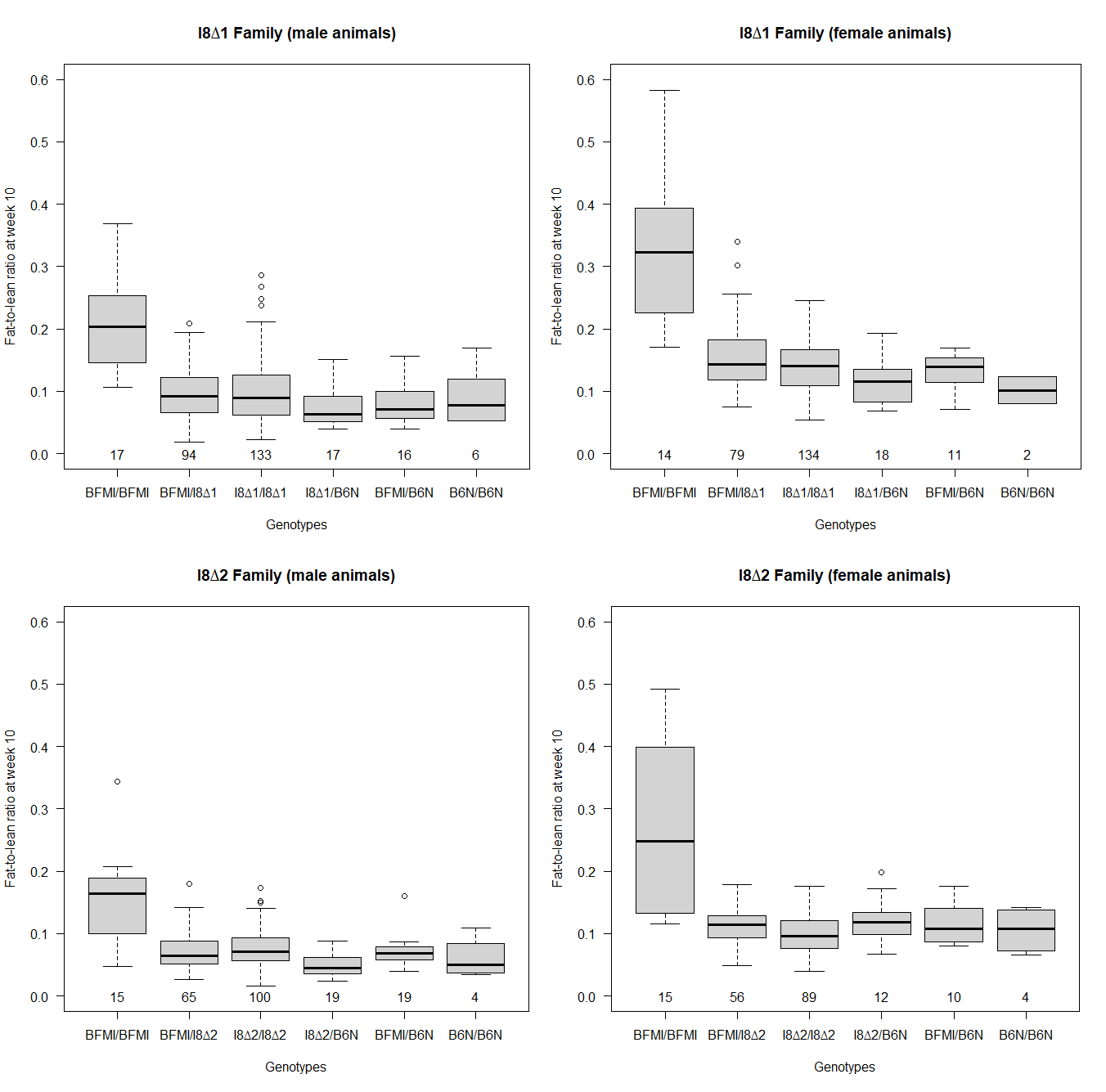


**Supplementary Figure 2: Uncorrected Fat and Lean percentages** A) fat % of male and female mice from family 1 (I8Δ1). B) lean % of male and female mice from family 1 (I8Δ1). C) fat % of male and female mice from family 2 (I8Δ2). D) lean % of male and female mice from family 2 (I8Δ2).


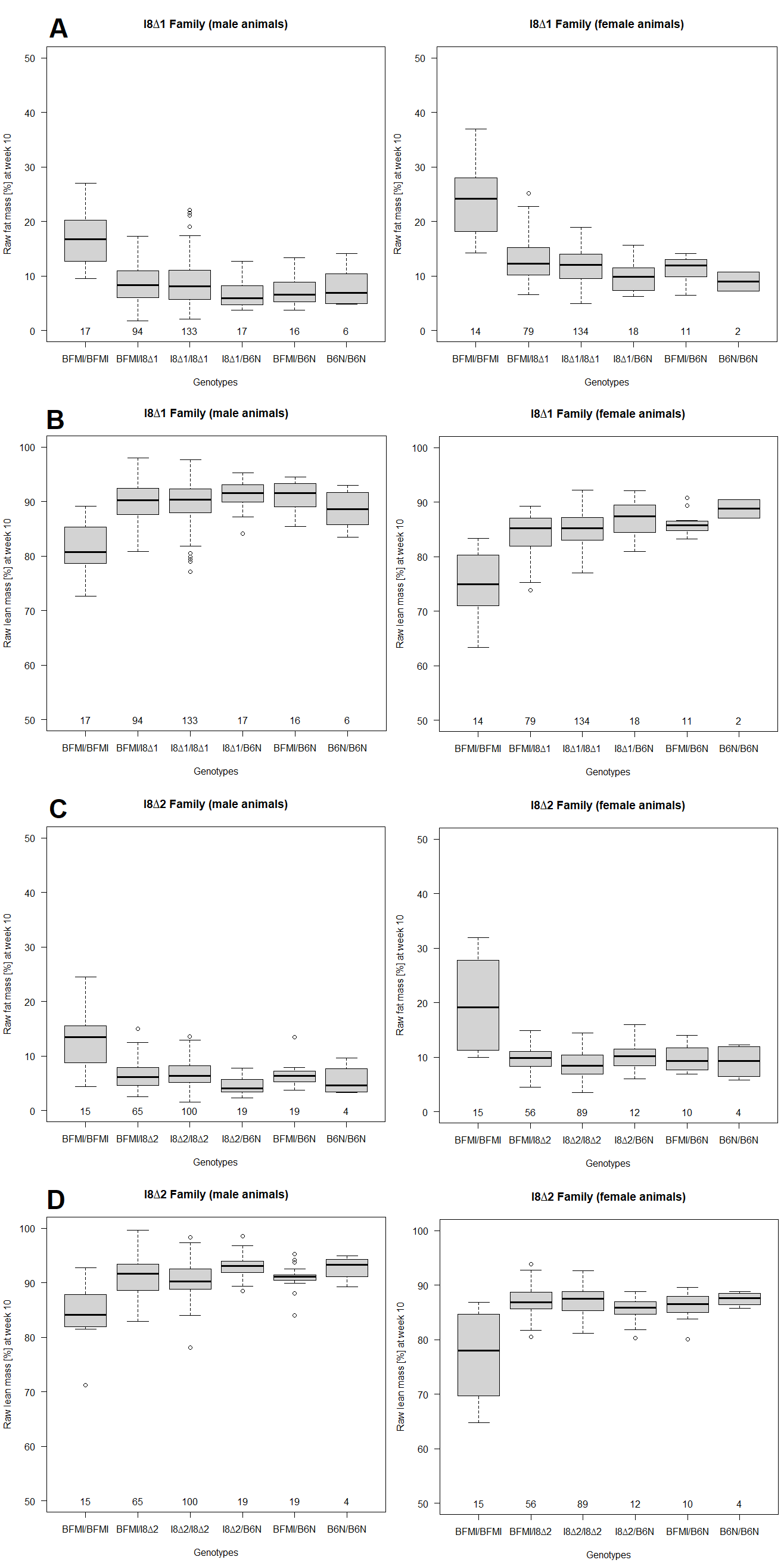

Supplement: Supplementary file 1 — Supplementary file1 (DOCX 156 kb) [file 335_2021_9938_MOESM1_ESM.docx]
